# Using Geopolymer Technology on Synthesizing Leucite Ceramics

**DOI:** 10.3390/polym13213621

**Published:** 2021-10-20

**Authors:** Yi-Che Hsieh, Wei-Hao Lee, Pin-Hsun Liao

**Affiliations:** 1 Department of Materials and Mineral Resources Engineering, National Taipei University of Technology, Taipei 10608, Taiwan; v2vvv222@gmail.com; 2 Institute of Mineral Resources Engineering, National Taipei University of Technology, Taipei 10608, Taiwan; a0912222490@gmail.com

**Keywords:** potassium-based geopolymers, leucite, ceramics

## Abstract

The aim of this study is to assess the process of synthesizing potassium-based geopolymers (KGL) into leucite ceramics with regard to five variables, namely, alkaline solution ratio (R), sintering time (S), calcining temperature (T), mixing time (M), and curing time (C). Under these conditions, the specimens were tested by the viscosity test, the mechanical properties test, X-ray diffraction (XRD), Fourier-transform infrared (FTIR) spectroscopy, and scanning electron microscopy (SEM) to understand the geopolymerization reactions and the characteristics of the KGL network. The results indicate that a KOH to K_2_O/SiO_2_ ratio of 1:1 promotes the reaction within metakaolin. XRD analysis of the KGL shows that, when the temperature is 1100 °C, the phase transforms into the leucite phase. Moreover, XRD analysis, mechanical properties, and FTIR all indicate improved characteristics when the curing time increases from 1 to 8 h. This might be attributed to the enhancement of the strong interaction between the matrix and the alkaline solution upon achieving adequate time to complete the geopolymerization process and forming a more stable three-dimensional structure. The formulation which formed the purest leucite phase consisted of: a 1:1 alkaline solution ratio, 10 min mixing time, 8 h curing time, 1200 °C calcining temperature, and 2 h sintering time.

## 1. Introduction

Geopolymers are made by mixing aluminosilicate-rich materials with alkaline solutions [1]. In addition to being obtained from natural ores, aluminosilicate materials are obtained from wastes, such as by-products, glass, fly ash, and blast furnace slag [2,3,4]. Alkaline solutions are mostly based on potassium and sodium, that not only promote geopolymerization to consolidate its colloid, but also have the function of balancing the charge of the aluminosilicate tetrahedron structures by their alkali metal cations [5]. The aluminosilicate material reacts with the alkaline solution, which dissolves the aluminum and silicon ions and constitutes aluminum and silicon gels on the surface of the powder particles. Then, the aluminum and silicon gels continuously polycondense, form into a continuous network structure, dehydrate, and form an amorphous or semi-crystalline, three-dimensional structure material [6,7].

Common geopolymer materials can be divided into four categories: (1) clay-based materials: kaolinite, metakaolin, sludge, etc. [8], (2) fly ash-based materials: coal fly ash, incineration fly ash, bottom ash, etc. [9], (3) calcium-based materials: ground granulated blast-furnace slag, electric arc-furnace slag, etc. [10], and (4) others: all sorts of waste glass, rice husk ash, red mud, etc. [11].

Metakaolin, formed by sintering kaolinite, has an uncharged octahedral structure. Under an alkaline environment, metakaolin can dissolve more aluminum, and silicon ions are provided with better geopolymer reactivity, enhancing the characteristics of the geopolymer [12]. He et al. [13] and Xu and Van Deventer [14] reveal that, after heat treatment, the phase transforms into an advanced reactivity amorphous phase, that progresses the overall background peak of the amorphous phase. Moreover, below 500–800 °C, the Al(OH)_3_ lamellar structures are bound to be progressing hydroxylation, thereby transforming the kaolinite into metakaolin within a disorder metastable phase; meanwhile, the lamellar structures are less dense, and both enhance the reaction rate in geopolymerization [15,16]. Due to metakaolin’s outstanding characteristics, it has been used in a wide range of ceramics, metals, coatings, polymers, and even composites [17,18,19,20,21,22,23].

Leucite is a potassium aluminum silicate mineral and has become a valuable material. It is used in formulations for metal-ceramic restorations to match the thermal contraction of the ceramic to that of the metal upon cooling [24]. Owing to their excellent strength and mechanical properties, leucite ceramics have been widely used in dental porcelains and structural ceramic materials [25,26,27,28,29]. However, to synthesize a pure leucite phase, the process requires extensive heat treatment at temperatures of 1500 °C [30], even with a long sintering period [31], which may have a disadvantage regarding the environmental impact. Thus, apart from the traditional methods, the eco-friendly geopolymer technology is based on the use of a potassium alkaline solution and metakaolin, which forms into a potassium-based geopolymer that provides another method for the energy consumption in the process. 

Bell et al. [32] explored the feasibility of utilizing metakaolin and an alkaline solution with a different ratio of potassium silicate solution and calcining between 950 and 1100 °C. The result shows that above 1000 °C, the phase transforms into the leucite phase. The behavior of potassium-based geopolymer between 950 and 1250 °C shows that a higher calcining temperature and a longer sintering time results in a higher degree of crystallization [33]. While controlling the Si/Al ratio of a potassium-based geopolymer, the characteristics were found to be a three-dimensional crystal growth mechanism [34]. Molten salt [35] and α-Al_2_O_3_ particle filler [36] can be added to a potassium-based geopolymer to increase the compressive strength and reduce the thermal shrinkage, respectively. 

Hitherto, studies have seldom focused on the effect of the alkaline solution ratio and ambient temperature curing time on potassium-based geopolymer leucite ceramics. Hence, in this study, specimens with five different factors were prepared, in order to determine the optimal process for further study.

## 2. Materials and Methods

### 2.1. Materials

**Figure 1:**
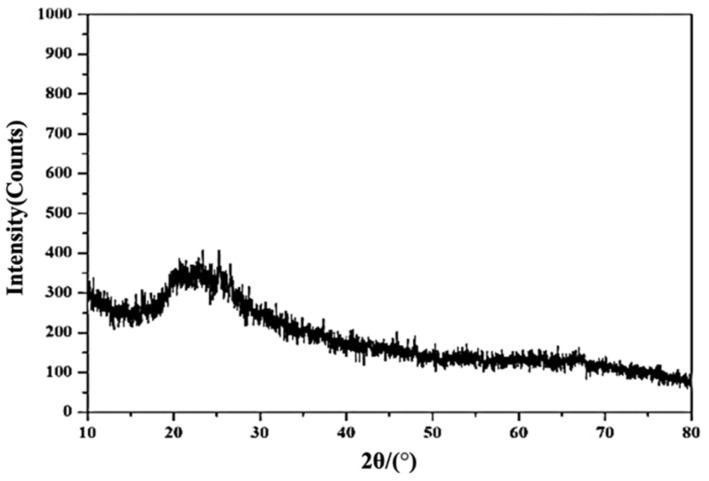
XRD result of metakaolin.

Metakaolin used in this study was purchased from Pei Long Enterprise Co. Ltd. (Changhua, Taiwan). The median particle diameter (D_50_) of metakaolin is 2.8 μm. Table 1 shows the chemical composition of metakaolin (mass%) as determined by X-ray fluorescence (XRF) spectrometry. The main components are SiO_2_ and Al_2_O_3_. X-ray diffraction (XRD) analysis and scanning electron microscopy (SEM) analysis of metakaolin are shown in Figure 1. According to Figure 1, the main mineral phase of metakaolin amorphous phase contained a large amount of glass. 

The alkaline solution was a mixture of potassium hydroxide solution and potassium silicate solution, according to various molar ratios of KOH to K_2_O/SiO_2_. The 86% industrial-grade potassium hydroxide was provided by Cheng Yi Chemical Co. Ltd. (Taipei, Taiwan) and the solutions were prepared with different concentrations of 10 M. Regarding the preparation of the potassium hydroxide solution, the first step was to dissolve KOH flakes in water for 20 min. Due to the heat and bubbles, the solution required a day to cool down to ambient temperature and defoam. The potassium silicate solution was provided by Rong Xiang Industrial Co. Ltd. (Taoyuan, Taiwan), and the model is RS-1, that consists of 23.8 wt.% SiO_3_ and 8.3 wt.% K_2_O.

### 2.2. Methods

In this study, five main influencing factors, namely, alkaline solution ratio (R), sintering time (S), calcining temperature (T), mixing time (M), and curing time (C), were investigated. Table 2, Table 3, Table 4, Table 5 and Table 6 show the details of the mixing proportions for the potassium-based geopolymer leucite (KGL).

First, the geopolymer pastes were mixed using metakaolin and different alkaline solutions’ formulas for 5 to 30 min, and the liquid/solid ratio was controlled at 1.2, then the viscosity of the slurry was measured. Simultaneously, the slurry was poured onto plates and cured at room temperature for 1 to 8 h. Second, all the samples were cured at 60 °C for 24 h, then cured at 100 °C for 24 h. Third, the hardened geopolymer sample was removed, crushed, and ground, and subsequently passed through 100-mesh (150-micron) sieves into powder. Fourth, the powder was filled into molds measuring Φ12.7 × 35 mm and then pressed with a hydraulic compression machine with a forming pressure of 150 kgf/cm^2^ into ingots. Finally, to obtain the stabilizing cubic leucite matrix composites, different sintering times and soaking temperatures were used. The sample was heated on a stove to 200 °C at a heating rate of 5 °C/min, then a soak time of 30 min, heated to 600 °C at a heating rate of 5 °C/min, then a soak time of 30 min, and then heated to 1000~1300 °C at a heating rate of 3 °C/min, then a soak time of 30 min to 4 h. After cooling, the KGL was cured at room temperature for analysis.

The viscosity of the geopolymer slurry was measured with a rotational automated viscometer (Brookfield RVF-100) under ambient temperature with rotors #5 and #6. The rotating speed and the factor are shown in Table 7. 

The viscosity was calculated as follows:

Reading×Factor=Viscosity (mPa·s)

Rockwell hardness testing was performed at a normal load of HR-15N in a ZHR8150LK model tester. The water absorption, porosity, and bulk-specific density tests, based on Archimedes’ principle, were conducted according to CNS (619 R3013) [37]. The specimens were subjected to XRD (Hitachi U-3310), FTIR spectroscopy (INVENIO), and SEM (ZEISS Gemini SEM500).

## 3. Results

### 3.1. Viscosity and Mechanical Properties

Table 8 shows the results of viscosity at different alkaline solution ratios. When specimens contain more potassium silicate, the viscosity is higher. This might be due to the higher potassium silicate proportion and too many silicon ions being produced and forming short Si-O or cyclic Si-O-Al bonds spontaneously. This type of bonding does not participate in geopolymerization, but increases the viscosity of the slurry [38]. However, when the ratio of potassium hydroxide increases, the slurry’s viscosity will also increase and develop the reaction of the geopolymer [39]. The impact of mixing time on viscosity is shown in Table 9. This indicates that, when the mixing time increases, viscosity will also increase. Due to the longer reaction time, the geopolymer has more time for condensation and polymerization, which may increase the viscosity.

The mechanical properties of KGL are shown in Table 8, Table 9, Table 10, Table 11 and Table 12. The apparent water absorption increase (from 0.4% to 18%) and porosity increase (from 0.3% to 32%), due to the increased KOH ratio, may result from the samples cast with higher molarity of KOH having greater absorption due to dependence upon cation type and pH [40]. When the calcining temperature rises, the bulk density increases from 2 to 3 g/cm^3^. This might be due to the higher temperature integrating the sample and crystallizing the inner structure and, as a consequence, enhancing the mechanical properties [33]. When the mixing time is increased, the porosity and water absorption decrease, due to the reaction rate increase, which can be verified by the increase of viscosity. Longer curing times are expected to improve the alkaline solution reaction with metakaolin, since K-based geopolymers require sufficient time to polycondense.

An increase in sintering time, calcining temperature, and curing time increase the hardness. R3 has a higher hardness under different alkaline solution ratios, which might correspond to the superior bulk density. It might also be due to the KOH loss tangent diminishing more rapidly, which shows that it favors activating the geopolymer structure, and probably establishing and forming larger oligomers or favoring the connectivity of a three-dimensional network [41]. Moreover, a decrease in porosity may be attributed to the fact that the alkaline activator in the mix has better reactivity with the raw materials, resulting in a denser microstructure, increased curing time, and decreased water absorption, due to the complete geopolymerization process [42]. This might be the reason for the increased hardness. 

### 3.2. Microstructures

#### 3.2.1. XRD

**Figure 2:**
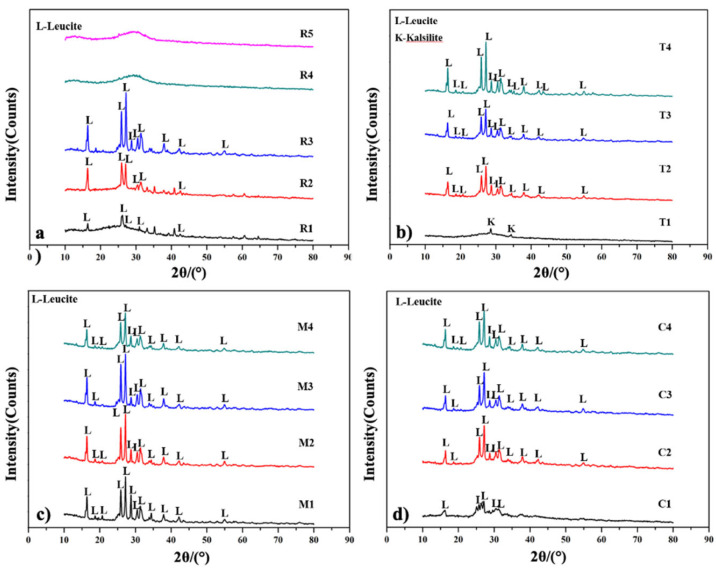
The results of XRD under different (**a**) alkaline solution ratios, (**b**) calcining temperatures, (**c**) mixing times, and (**d**) curing times.

As shown in Figure 2a, when the potassium hydroxide exceeds the potassium silicate, the phase of KGL is amorphous. In contrast, when the potassium silicate is similar to or exceeds the potassium hydroxide, the leucite phase will appear. However, if potassium silicate alone is added, the intensity of the leucite is low. Therefore, the optimal alkaline ratio is R3, where the potassium hydroxide to potassium silicate ratio is 1:1.

Figure 2b shows the result of XRD at different calcining temperatures. The main phase of T1 was Kalsilite: when the calcining temperature increased to 1100 °C, the specimens crystallized into a leucite phase. Identical phenomena have been reported elsewhere [32,33,43,44,45] and might also present cubic leucite and tetragonal leucite in 26.53°2θ and 27.28°2θ, respectively [34,46].

Figure 2c details the XRD pattern under different mixing times, and most of the phases were leucite crystallization. M1 shows lower intensity, which might be due to an insufficient geopolymer reaction. The lower intensity of M4 might be due to the integrity geopolymerization process being destroyed by excessive mixing.

Figure 2d shows representative XRD patterns from distinct curing times, consistent with the mechanical property results. The intensity of specimens C1 to C4 increased due to the increased curing time, which enables the specimens to form into a complete three-dimensional network.

#### 3.2.2. FTIR

**Figure 3:**
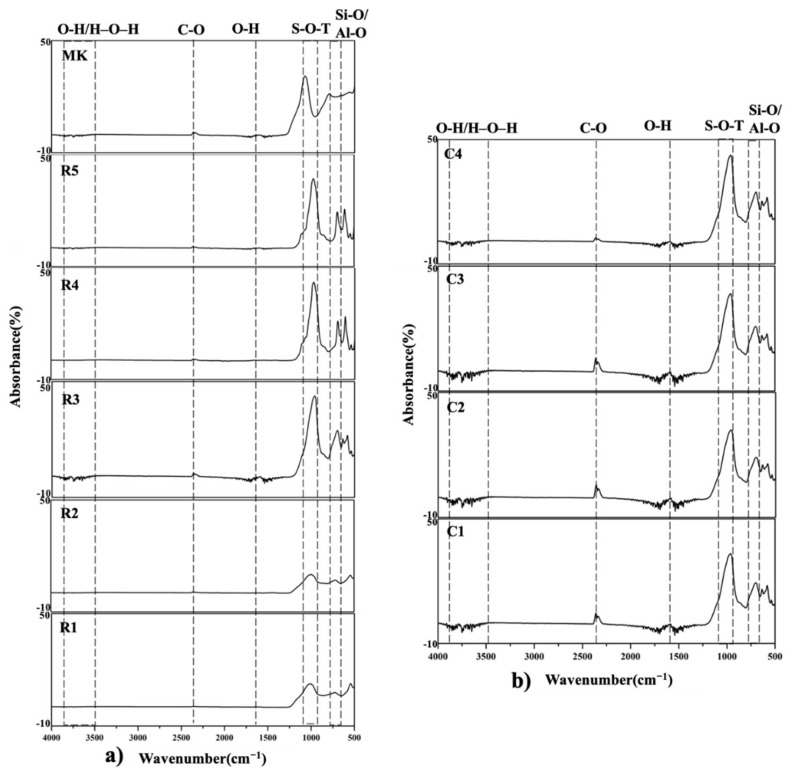
FTIR analysis of KGL prepared with different (**a**) alkaline solution ratios and (**b**) curing times.

Figure 3a shows the FTIR analysis results for various alkaline solution ratios. Apparent changes in the molecular and bond structure were observed in KGL and metakaolin. The main peak of metakaolin was at 1090 cm^−1^, assigned to the stretching of the Si-O bond in SiO_2_ [47]. The peak observed at 810 cm^−1^ corresponds to a typical AlIV [48] that completely disappears after forming into geopolymer samples. The absorption bands at 2352 cm^−1^ represent the vibrations of the C-O bond, attributed to the presence of carbonates, that homogeneously dissolved at the molecular level and are not present in fluid inclusions. In short, the C-O bonds adsorbed to a glassy phase in the form of CO_2_ molecules [49,50]. Specimens R4 and R5, which contained a higher ratio of potassium hydroxide, show a new band at about 1103 cm^−1^ for KOH [51]. The band at approximately 1090 cm^−1^ shifts to 1035–950 cm^−1^, coinciding with the Si-O-T bond and indicating structural changes in metakaolin. This could refer to aluminosilicate framework structures, which are assigned to internal vibrations of the Si-O-Si and the Si-O-Al bonds [52,53]. The shift changes in the Al content of the formed geopolymeric gels have been assigned by many authors [51,54,55,56]. 

Kljajević et al. [57] indicate that the observed shift of the Si-O-T bond appears to be due to the reference geopolymer having a modestly regulated structure. Whilst annealing increases bond strength, it might improve mechanical properties, similar to ceramics that are formed by annealing. Moreover, the primary Si-O-T band at approximately 980 cm^−1^ can be considered a Q2 position shift [58,59]. The variation reveals a polycondensation reaction, characteristic of the formation of a specific network, and might point to the formation into a complete three-dimensional network. The bands in the range of 850–600 cm^−1^ are related to the ring vibrations of the Si-O bonds of the silicate network [60,61], and the Al-O stretching vibrations might be the bending mode of AlO_6_ octahedra [62,63,64].

Figure 3b shows the FTIR analysis results under different curing times. The bands at around 3430^−1^ and a band at 1630 cm^−1^ are characteristic of the stretching and bending vibrations of O-H and H-O-H bonds from the physically adsorbed water [65,66,67,68]. When the curing time is increased, the O-H/H-O-H bond vibrations decrease. That may account for the alkaline solution having adequately reacted with metakaolin at the same time that dehydration started, thus enhancing the geopolymerization. This might correspond to the intensity increase in Figure 2d and the density increase in Table 11.

#### 3.2.3. SEM

**Figure 4:**
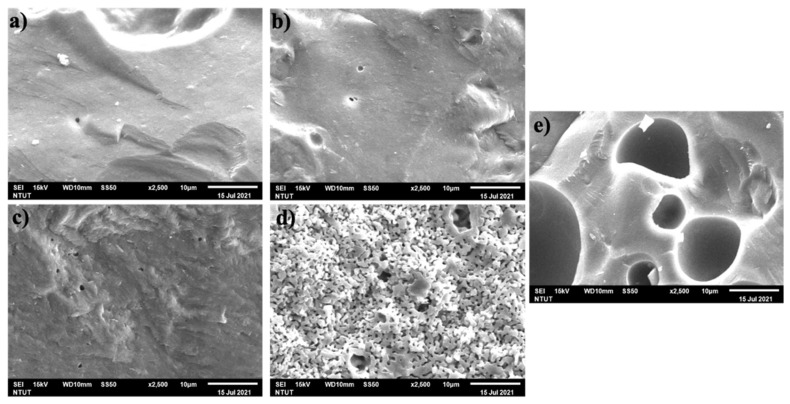
SEM images (×2500) of (**a**) R1, (**b**) R2, (**c**) R3, (**d**) R4, and (**e**) R5.

Figure 4 shows SEM images under different alkaline solution ratios. There is a significant change in microstructure associated with changing the alkaline solution ratio. Figure 4a–c show a few pores on the surface that can correspond to the results of porosity in Table 7. When the proportion of KOH increases, the microstructures contain obvious pores that can be seen in Figure 4d,e, and which indicate similar results within the mechanical properties. 

**Figure 5:**
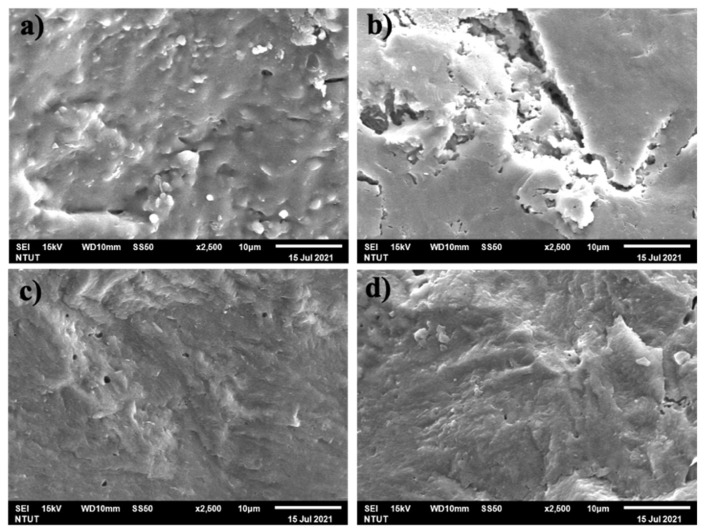
SEM images (×2500) of (**a**) T1, (**b**) T2, (**c**) T3, and (**d**) T4.

SEM images under different calcining temperatures are shown in Figure 5. Although there are prominent peaks of leucite in the X-ray results for T2, T3, and T4, none of the leucite crystals could be directly observed in Figure 5, similar to the results of Xie et al. [33]. There were scarcely any microcracks in the surface of Figure 5c,d, compared with Figure 5a,b. As shown in Figure 5a–d, these precipitates coarsened substantially after KGL was heated from 1000 to 1300 °C. Coarsening might form structural densification and develop the surface into a glassy texture, which is generally observed as a consequence of dehydration of the geopolymeric products formed and the structural changes generated [32,69].

## 4. Conclusions

The conclusions from this study, regarding synthesizing metakaolin geopolymer leucite KGL, can be summarized as follows:

KOH to K_2_O/SiO_2_ ratio and mixing time can significantly influence the viscosity and the mechanical properties. A high KOH to K_2_O/SiO_2_ ratio results in high porosity and low bulk density. Scenario R3, where the KOH to K_2_O/SiO_2_ ratio was 1:1, showed the optimal viscosity, bulk density, and hardness under different alkaline solution ratios and mixing times.

X-ray diffraction suggested that the KOH to K_2_O/SiO_2_ ratio and curing time significantly affect the intensity, while intensity is less sensitive to mixing time. On the other hand, the KGL specimens started to form into leucite peaks when heated at >1100 °C.

The molecular and bond structures of metakaolin and KGL have apparent changes. A longer curing time might cause the specimens to adequately react with metakaolin; meanwhile, dehydration enhances the geopolymerization.

Scanning electron microscopy suggested that, when the calcining temperature increases, KGL develops a surface with a glassy texture, resulting in a denser structure, which seems likely to be associated with the dehydration of potassium.

## Figures and Tables

**Table 1:** Chemical composition of metakaolin.

Oxide (wt.%)	Metakaolin
SiO_2_	49.8
Al_2_O_3_	19.3
TiO_2_	10.2
Fe_2_O_3_	6.3
K_2_O	2.4
CaO	2.2
Others	6.8
L.O.I	3.0

**Table 2:** Mixing proportion at different alkaline solution ratios.

Specimen Label	KOH	K_2_O/SiO_2_	Sintering Time (h)	Temperature (°C)	Mixing Time (min)	Curing Time (h)
R1	0	1	2	1200	10	8
R2	0.25	0.75				
R3	0.5	0.5				
R4	0.75	0.25				
R5	1	0				

**Table 3:** Mixing proportion at different sintering times.

Specimen Label	KOH	K_2_O/SiO_2_	Sintering Time (h)	Temperature (°C)	Mixing Time (min)	Curing Time (h)
S1	0.5	0.5	0.5	1200	10	8
S2			1			
S3			2			
S4			4			

**Table 4:** Mixing proportion at different calcining temperatures.

Specimen Label	KOH	K_2_O/SiO_2_	Sintering Time (h)	Temperature (°C)	Mixing Time (min)	Curing Time (h)
T1	0.5	0.5	2	1000	10	8
T2				1100		
T3				1200		
T4				1300		

**Table 5:** Mixing proportion at different mixing times.

Specimen Label	KOH	K_2_O/SiO_2_	Sintering Time (h)	Temperature (°C)	Mixing Time (min)	Curing Time (h)
M1	0.5	0.5	2	1200	5	8
M2					10	
M3					15	
M4					30	

**Table 6:** Mixing proportion at different curing times.

Specimen Label	KOH	K_2_O/SiO_2_	Sintering Time (h)	Temperature (°C)	Mixing Time (min)	Curing Time (h)
C1	0.5	0.5	2	1200	10	1
C2						2
C3						4
C4						8

**Table 7:** Coefficients of factor.

Rotor Speed	Rotor						
	1	2	3	4	5	6	7
10	10	40	100	200	400	1000	4000
20	5	20	50	100	200	500	2000
50	2	8	20	40	80	200	800
100	1	4	10	20	40	100	400

**Table 8:** The results of viscosity and mechanical properties at different alkaline solution ratios.

Specimen Label	Viscosity (MPa.s)	Water Absorption (%)	Porosity (%)	Bulk Density (g/cm^3^)	Rockwell Hardness (GPa)
R1	44,000	0.4	0.3	2.52	2.62
R2	12,000	1	2	2.34	2.67
R3	5250	1	2	3.21	6.02
R4	9000	18	32	2.02	none
R5	9850	18	32	1.73	none

**Table 9:** The results of viscosity and mechanical properties at different mixing times.

Specimen Label	Viscosity (MPa.s)	Water Absorption (%)	Porosity (%)	Bulk Density (g/cm^3^)	Rockwell Hardness (GPa)
M1	3250	13	25	2.06	4.04
M2	5250	1	2	3.21	6.02
M3	14,500	1	2	2.55	4.98
M4	24,000	1	2	2.39	4.85

**Table 10:** The results of viscosity and mechanical properties at different sintering times.

Specimen Label	Viscosity (MPa.s)	Water Absorption (%)	Porosity (%)	Bulk Density (g/cm^3^)	Rockwell Hardness (GPa)
S1	-	1	3	2.34	5.18
S2	-	1	3	2.40	5.03
S3	5250	1	2	3.21	6.02
S4	-	1	2	3.38	6.15

**Table 11:** The results of viscosity and mechanical properties at different calcining temperatures.

Specimen Label	Viscosity (MPa.s)	Water Absorption (%)	Porosity (%)	Bulk Density (g/cm^3^)	Rockwell Hardness (GPa)
T1	-	2	4	2.12	4.41
T2	-	2	2	2.82	4.81
T3	5250	1	2	3.21	6.02
T4	-	0.7	1	3.46	5.54

**Table 12:** The results of viscosity and mechanical properties at different curing times.

Specimen Label	Viscosity (MPa.s)	Water Absorption (%)	Porosity (%)	Bulk Density (g/cm^3^)	Rockwell Hardness (GPa)
C1	-	2	7	2.28	3.805
C2	-	1	5	2.47	3.88
C3	-	1	4	3.08	4.51
C4	5250	1	2	3.21	6.02
